# Triterpenoids and polysaccharide peptides-enriched *Ganoderma lucidum*: a randomized, double-blind placebo-controlled crossover study of its antioxidation and hepatoprotective efficacy in healthy volunteers

**DOI:** 10.1080/13880209.2017.1288750

**Published:** 2017-02-09

**Authors:** Hui-Fang Chiu, Hui-Yu Fu, Yan-Ying Lu, Yi-Chun Han, You-Cheng Shen, Kamesh Venkatakrishnan, Oksana Golovinskaia, Chin-Kun Wang

**Affiliations:** a Department of Chinese Medicine, Taichung Hospital, Ministry of Health and Well-being, Taichung, Taiwan, Republic of China;; b School of Nutrition, Chung Shan Medical University, Taichung City, Taiwan, Republic of China;; c Department of Neurology, Chung Shan Medical University, Taichung City, Taiwan, Republic of China;; d School of Health Diet and Industry Management, Chung Shan Medical University, Taichung City, Taiwan, Republic of China;; e Department of Food Science, ITMO University, Saint-Peterburg, Russia

**Keywords:** Oxidative stress, ultrasonic examination, hepatic markers, fatty liver

## Abstract

**Context:***Ganoderma lucidum* (Leyss: Fr) Karst. (Polyporaceae) is an oriental medicinal fungus, commonly used in traditional Chinese medicine (TCM) for treating various condition or diseases such as hypertension, hyperglycaemia, hepatitis and cancer.

**Objective***:* The current study examines whether triterpenoids and polysaccharide-enriched *G. lucidum* (GL) influence antioxidation and hepatoprotective efficacy by suppressing oxidative stress.

**Materials and methods:** Forty-two healthy subjects (22 male and 20 female) were recruited and segregated into two groups as experimental or placebo and requested to intake GL (*n* = 21) or placebo (*n* = 21) capsule (225 mg; after lunch or dinner) for six consecutive months and *vice versa* with one month washout period in between. The anthropometric analysis and biochemical assays, as well as abdominal ultrasonic examination were performed.

**Results:** Consumption of GL substantially improved (*p* < 0.05) the total antioxidant capacity (TEAC; 79.33–84.04), total thiols and glutathione content (6–8.05) in plasma as well as significant (*p* < 0.05) enhanced the activities of antioxidant enzymes. Whereas, the levels of thiobarbituric acid reactive substances (TBARS; 3.37–2.47), 8-hydroxy-deoxy-guanosine (8-OH-dG; 15.99–11.98) and hepatic marker enzymes (glutamic-oxaloacetic transaminase; GOT and glutamic-pyruvic transaminase; GPT) were concomitantly reduced (42 and 27%) on treatment with GL. Furthermore, the abdominal ultrasonic examination in GL subjects displayed a notable alteration on hepatic condition by reversing from mild fatty liver condition (initial) to normal condition.

**Discussion and Conclusion:** The outcome of the present intervention demonstrated the antioxidation, anti-aging and hepatoprotective nature of GL by effectively curbing oxidative stress.

## Introduction

In recent times, traditional Chinese medicine (TCM) is becoming more popular worldwide, owing to its holistic and synergic effect with few adverse events. TCM is a well accredited alternative therapy for treating various diseases or disorders. *Ganoderma lucidum* (Leyss: Fr) Karst. (Polyporaceae) (GL), a dark woody texture, glossy mushroom commonly called ‘Lingzhi’, is a well-known drug used in TCM for treating various metabolic disorders such as cancer, cardiovascular disease and diabetes (Deepalakshmi & Mirunalini 2011; Wachtel-Galor et al. 2004a) as well as to prolong the lifespan and hence, called the ‘mushroom of longevity’ (Wang et al. 2006). GL is rich with several types of triterpenoids, steroids, peptides, nucleotides and polysaccharides. However, most of the pharmacological properties of GL are influenced by triterpenoids and polysaccharides (Sheena et al. 2003; Yang 2005). Recently, Li et al. (2016) hinted that the quality of GL is directly proportionate to the contents of triterpenoids and polysaccharides. Some major triterpenoids found in GL are ganoderic (highly oxygenated C_30_ lanostane), lucidenic and ganodermic acid (Boh et al. 2007). Some of the therapeutic effects of triterpenoids and polysaccharides of GL are antioxidant, anti-inflammatory, hypoglycemic, anticancer, anti-hypercholesterolaemic, antimicrobial and anti-hepatotoxin effects (Bao et al. 2002; Gao & Zhou 2009; Wu et al. 2016). Moreover, GL is rich in essential amino acids such as lysine, histidine, phenylalanine and leucine (Rubel et al. 2011), which might upregulate the gene involved in antioxidative enzymes and thereby maintain the balance between free radical production and antioxidant status.

Increasing lines of evidence have shown that excessive generation of reactive oxygen species (ROS) is the crucial factor for contributing various disorders via triggering oxidative stress (Rattan 2006). Oxidative stress is nothing but an imbalance between oxidants and antioxidants. Oxidative stress may result in cellular damage by attacking macromolecules such as carbohydrates, lipids, proteins and nucleic acids, especially DNA (Olmez & Ozyurt 2012). If this cellular damage persists for a longer time, it might end up in premature aging (Ko et al. 2008) and finally ends up in several chronic diseases (Lai et al. 2008). Hence, the usage of some natural antioxidants like GL (triterpenoids and polysaccharides) might lower the ROS generation and thereby render a protection from various degenerative diseases and slower aging process. Ample amount of animal studies had shown the antioxidant, hepatoprotective and longevity activity of GL (Han et al. 2006; Jia et al. 2009; Pan et al. 2013), however, no or less clinical trial was carried out. Although, GL is a renowned Chinese Medicine recommended for promoting health status and for longevity and hepatoprotection, but still the scientific evidence associated with a clinical trial for anti-aging and hepatoprotective effects of GL in long-term phase is yet to be elucidated. Moreover, free radicals are the initiator of the aging process and hence, it is reasonable to determine whether GL probably influences anti-aging and hepatoprotective efficacy by suppressing oxidative stress in a double-blind placebo-controlled randomized crossover clinical trial. Hence, this randomized crossover clinical trial was aimed to examine, the antioxidative (anti-aging) and hepatoprotective efficacy of GL enriched with triterpenoids and polysaccharide in healthy volunteers.

## Materials and methods

### GL and placebo capsule

GL capsule was provided by Double Crane, Enterprise Co., Ltd. Taiwan. Each capsule (225 mg) contains 7% triterpenoid-ganoderic acid (A, B, C, C_5_, C_6_, D, E and G), 6% polysaccharide peptides with few essential amino acids and trace elements. Placebo capsule contains 90% of starch and 10% of GL residues and appears similar to GL capsules.

### Subjects

Forty-two healthy middle-aged (40?54 years-of-age) volunteers with mild liver dysfunction (Elevated GOT and GPT as well as fatty liver) were recruited for the current intervention through posters or advertisements. This randomized, placebo-controlled crossover study was carried out at Chung Shan Medical University Hospital, according to the Declaration of Helsinki and Good Clinical Practice (GCP). The study protocol was approved by the Institutional Review Board (IRB) of Chung Shan Medical University Hospital, Taichung, Taiwan (CSMUH No. 03043). Each subject was informed about the study details and was asked to sign consent. The exclusion criteria for this study included subjects with uncontrolled diabetes, mental illness or depression, kidney, or heart disease, pregnancy, and breast-feeding woman, as well as with the history of stroke. Subjects were instructed not to intake any medications or supplements throughout the experimental period.

A physical (anthropometric measurements) and basic biochemical (GOT, GPT) examination were performed to 42 subjects at the beginning of the intervention and they were randomly divided into two groups with 21 subjects in each (11 male and 10 female) who received one GL or placebo capsules (after lunch or dinner respectively) daily for 6 months and *vice versa* with one month of washout period in between. During the initial, third, sixth months anthropometric measurements were performed. Researcher who treated or checked the subjects were forbidden from the subject’s treatment allocation (double-blind). Based on subject’s record, the average percentage intake of GL or placebo was noted as 85.11%. Three female (due to an unwillingness to cooperate) gave up from the study and thus concluded with 39 subjects.

### Blood collection and abdominal examination

During baseline (initial), midpoint (third month) and end of each intervention period (sixth month), fasting blood samples were collected in an EDTA-coated vacuum tube, and the plasma was separated by centrifuging at 1500 *g* (Supercentrifuge; 1K15). Separated blood samples after deducting the intermediate film, settling part were washed with isotonic saline and centrifuged at 1500 *g* to get erythrocytes and used for assaying antioxidant enzymes. All the samples were stored at −80 °C until analysis. At the initial and sixth month the abdominal ultrasonic examination was performed by Gastroenterologist Dr Chen Ziyan in Chung Shan Medical University Hospital, Taichung, Taiwan.

### Determination of various oxidative indexes

Trolox-equivalent antioxidant capacity (TEAC) by Arnao et al. (1990) and Miller et al. (1993), TBARS by Draper and Hadley (1990), total thiols by Hamva et al. (1992) and Hu (1994) as well as glutathione content by Halliwell and Gutteridge (1990) in plasma were determined by the methods mentioned above. The levels of plasma 8-hydroxy-deoxy-guanosine (8-OH-dG) were determined by using high sensitive 8-OH-dG ELISA kits (Cosmo Bio USA Inc, Carlsbad, CA) based on manufacturer’s protocol.

### Assay of erythrocyte antioxidants and hepatic marker

Superoxide dismutase (SOD) activity was determined with the Ransod SD 125 kit (Randox Labs, Crumlin, UK), Glutathione peroxidase (GPx), Glucose-6-phosphate dehydrogenase (G-6-PDH), and glutathione reductase (GR) activities were determined with the Ransel RS 504 kit and G6p dh assay kit (Randox Labs, Crumlin, UK) and catalase (CAT) activity was assayed using the method by Aebi (1984). Erythrocyte protein contents were determined by BCA kit from Thermo Fisher Scientific (Waltham, MA). The plasma glutamic oxaloacetic transaminase (GOT) and glutamic pyruvic transaminase (GPT) were assayed by commercial kits from AppliedBio (Hercules, CA).

### Statistical analysis

The data were expressed as a mean ± standard deviation (SD). The paired *t* test was applied to compare the difference in the same group (baseline vs. end of treatment), and the Student’s *t* test was used to compare the difference between the experimental (GL) and control (placebo) groups and the variables were analyzed via one-way ANOVA (parametric) using Statistical Package for the Social Sciences (SPSS) version 21.0 (Chicago, IL). A value of *p* < 0.05 was counted statistically significant.

## Results and discussion

**Figure 1.**
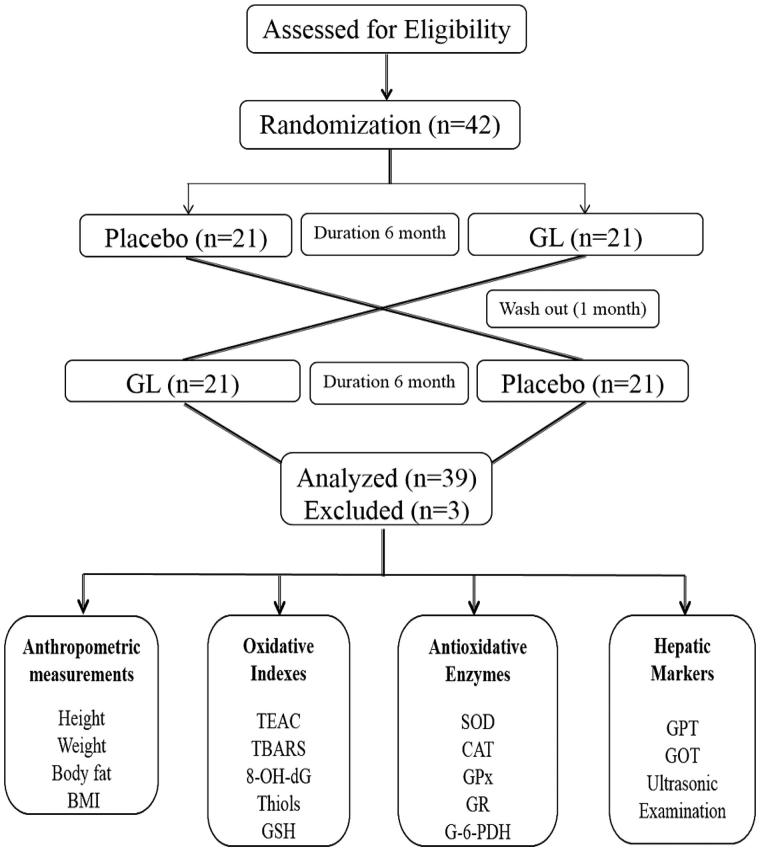
Flow chart of present study.

The purpose of this randomized crossover clinical trial was to probe, whether triterpenoids and polysaccharide-enriched GL influence antioxidative (anti-aging) and hepatoprotective efficacy via attenuating overproduction of free radicals and resultant oxidative stress. Several reports have indicated the direct link between oxidative stress and premature aging through increased cellular damage in the form of lipid peroxidation (Ko et al. 2008). Hence, well-known antioxidant such as GL was preferred to combat against free radical-induced cellular damage especially hepatocytes. This study is the first long-term, double-blind placebo-controlled randomized crossover clinical trial with GL as well as the correlation between the free radicals with hepatotoxicity and the aging process. Moreover, the crossover experimental design was used (as each subject will intake both GL and placebo capsule) to eliminate confounding factors and ideal design for pilot studies. During the preliminary phytochemical analysis, the two major active components, triterpenoids, and polysaccharides in GL were assessed by both qualitatively (Liebermann-Burchard’s method) and quantitatively which indicated 6.8 and 5.5%, respectively (data not shown), that are almost equal to the composition given by Double Crane, Enterprise Co., Ltd. Moreover, triterpenoids are separated by using HPLC technique to confirm the quality (ganoderic acid, data not shown). A flow chart of the current study is illustrated in Figure 1.

The anthropometric parameters in GL and placebo-treated healthy subjects are exemplified in Table 1. During baseline of GL and the placebo group the height, weight, body fat and body mass index (BMI) were in normal range, indicating the involvement of healthy subjects. Experimental subject who ingested GL capsule for 6 months did not show any notable changes in any of the values of anthropometric parameters, thereby confirming that GL was devoid of any undesirable events, even though GL were supplemented for 6 consecutive months.

**Table 1.** The anthropometric parameters in GL and placebo-treated healthy subjects.

	Group	Height (cm)	Weight (Kg)	Body fat (%)	BMI (kg/m^2^)
Baseline	GL	165.24 ± 8.55^a^	63.63 ± 10.75^a^	26.06 ± 4.68^a^	23.22 ± 2.93^a^
	Placebo	164.78 ± 10.11^a^	63.75 ± 10.89^a^	25.37 ± 4.28^a^	23.26 ± 2.97^a^
Third month	GL	165.24 ± 8.55^a^	62.98 ± 10.98^a^	25.79 ± 4.20^a^	22.95 ± 3.02^a^
	Placebo	164.78 ± 10.11^a^	63.55 ± 11.45^a^	26.23 ± 4.00^a^	23.19 ± 3.18^a^
Sixth month	GL	165.24 ± 8.55^a^	63.36 ± 11.01^a^	25.91 ± 4.54^a^	22.97 ± 3.31^a^
	Placebo	164.78 ± 10.11^a^	63.18 ± 11.09^a^	26.43 ± 4.47^a^	23.05 ± 3.06^a^

Values were expressed as means ± SD (*n* = 39). Data within the same column of each group sharing different superscript letters were significantly different (*p* < 0.05).

BMI: body mass index; GL: *Ganoderma lucidum*.

Increasing lines of evidence have indicated that excessive generation of reactive oxygen species (ROS) is the important factor for contributing various chronic diseases and premature aging via triggering oxidative stress (Rattan 2006). The human body was well equipped with the antioxidant defence system to encounter those excessive ROS (Kamesh & Sumathi 2014). Table 2 shows the levels of plasma TEAC, total thiols and GSH in GL and placebo-treated subjects. TEAC, total thiols and GSH levels (oxidative markers) were improved (*p* < 0.05) progressively from baseline to sixth month, upon consumption with GL. Many studies demonstrated that several types of triterpenoids and polysaccharides in GL might act as antioxidants by effectively donating its electron or proton and thereby halted free-radical generation (Boh et al. 2007; Yang et al. 2007). Wachtel-Galor et al. (2004b) pointed out that, plasma antioxidant levels were substantially improved after the supplementation with a commercial GL product.

**Table 2.** Various plasma oxidative indexes in GL and placebo-treated healthy subjects.

	Group	TEAC (%)	TBARS (μM/L)	8-OH-dG (pg/mL)	Thiols (mM/mL)	GSH (μM/L)
Baseline	GL	79.33 ± 4.95^a^	3.37 ± 0.47^a^	15.99 ± 2.39^a^	0.19 ± 0.04^b^	6.00 ± 1.07^b^
	Placebo	80.70 ± 5.04^a^	3.26 ± 0.44^a^	14.70 ± 3.00^a^	0.21 ± 0.06^a^	6.90 ± 1.48^a^
Third month	GL	82.93 ± 3.087^a^	3.28 ± 0.81^a^	14.49 ± 2.72^a^	0.20 ± 0.05^b^	7.30 ± 1.66^a^
	Placebo	80.97 ± 3.98^a^	3.32 ± 0.73^a^	15.19 ± 2.99^a^	0.20 ± 0.07^a^	6.66 ± 1.63^a^
Sixth month	GL	84.04 ± 3.74^b^	2.47 ± 0.68^b^	11.98 ± 1.79^b^	0.28 ± 0.05^a^	8.05 ± 1.42^a^
	Placebo	80.24 ± 3.79^a^	3.30 ± 0.88^a^	15.35 ± 3.07^a^	0.19 ± 0.06^a^	6.63 ± 1.39^a^

Values were expressed as means ± SD (*n* = 39). Data within the same column of each group bearing different superscript letters were significantly different (*p* < 0.05).

At present, very few universally accepted ubiquitous oxidative stress biomarkers are identified, out of which TBARS and 8-OH-dG are the major biomarkers. Several studies point out the strong correlation existed between pre-matured aging and elevated oxidative stress biomarkers (Pandey & Rizvi 2010). During oxidative stress condition, the outer plasma membrane is damaged (polyunsaturated fatty acids-lipid peroxidation) by excessive free radicals, which contributes to increased cellular damage and elevated TBARS. Once the lipid peroxidation process progress, it tends to damage the inner cellular membrane of mitochondria and the nucleus and finally damages DNA, that trigger the excessive production of 8-OH-dG by-products (Valavanidis et al. 2009). The levels of oxidative stress markers such as TBARS and 8-OH-dG are shown in Table 2. During the baseline, levels of TBARS and 8-OH-dG were found as 3.37 ± 0.47 μM/L and 15.99 ± 2.39 pg/mL, but after administration with GL for 6 months (*p* < 0.05) the levels were considerably suppressed to 2.47 ± 0.68 μM/L and 11.98 ± 1.79 pg/mL. The present outcome clearly inferred the beneficial effect of GL by lowering oxidative stress marker. Our results are in corroboration with the results by Pan et al. (2013), who proved that treatment with GL extract significantly reduced the levels of both 8-OH-dG and lipid peroxidation products in mice model, due to its free radical scavenging activity. Furthermore, Lee et al. (2001), hinted that GL supplementation significantly lowers the lipid peroxidation and prevented DNA damage and can be used as an anti-aging agent.

An antioxidant is a substance that significantly suppresses or inhibits oxidation of another substance. Usually, there is a balance between ROS production and antioxidant defence system that is indispensable for normal metabolism. Therefore, our cells produce some endogenous antioxidants such as SOD, CAT, GPx, and GSH to suppress excessive free radicals (Wu et al. 2016). The erythrocyte antioxidant enzymes such as SOD, CAT, G-6-PDH, GPx and GR in the GL and placebo group are illustrated in Table 3. No notable changes were observed in the placebo-treated group from baseline to sixth month. However, GL-treated subjects exhibit a substantial escalation in the levels of SOD, CAT, GPx and G-6-PDH on a comparison between baseline and the end of the treatment (sixth month). Meanwhile, GR levels in GL-treated subjects did not show any significant changes, but the increasing trend was noted from baseline to sixth month. These increase in antioxidant enzymes in GL was primarily contributed by ganoderic acid that could activate the nuclear factor erythroid 2-related factor 2 (Nrf2), which in turn initiate the expression of antioxidant genes (*SOD*, *CAT* and *GPx*) and thereby enhanced the antioxidant enzyme levels (Ko et al. 2008). Also, the treatment with *G. lucidum* maintained normal levels of *SOD*, *CAT* and *GSH* (Jia et al. 2009). GL is rich in essential amino acids such as lysine, histidine, phenylalanine and leucine which are directly involved in the synthesis of various endogenous antioxidative enzymes such as SOD, CAT, GSH and thereby suppress the hyper-generation of free radical (Deepalakshmi & Mirunalini 2011). Furthermore, some trace elements (minerals) such as calcium, zinc, sodium, potassium, magnesium and selenium present in GL probably influence free radical quenching ability, by acting as cofactors to antioxidant enzymes (Iranzo 2011). Ko et al. (2008) indicated that triterpenoids such as GA-B,-C2 and –G of GL possess anti-aging activity by enhanced antioxidant property.

**Table 3.** Erythrocyte antioxidative enzymes in GL and placebo-treated healthy subjects.

	Group	SOD (IU/g Hb)	CAT (IU/g Hb)	G-6-PDH (IU/g Hb)	GPx (IU/g Hb)	GR (IU/g Hb)
Baseline	GL	1155.98 ± 150.11^c^	246.26 ± 28.08^b^	11.99 ± 1.99^b^	13.16 ± 1.71^b^	4.00 ± 0.61^a^
	Placebo	1143.95 ± 170.14^a^	245.83 ± 32.43^a^	11.83 ± 2.11^b^	12.64 ± 1.43^a^	3.95 ± 0.64^a^
Third month	GL	1244.73 ± 149.46^b^	268.87 ± 28.22^ab^	12.40 ± 2.26^b^	14.39 ± 1.20^b^	4.29 ± 0.66^a^
	Placebo	1141.25 ± 155.46^a^	244.86 ± 31.80^a^	11.93 ± 2.19^a^	13.07 ± 1.53^a^	3.99 ± 0.63^a^
Sixth month	GL	1385.63 ± 139.01^a^	279.21 ± 26.18^a^	13.56 ± 2.11^a^	15.44 ± 1.17^a^	4.53 ± 0.68^a^
	Placebo	1144.60 ± 150.73^a^	242.97 ± 28.32^a^	11.94 ± 2.03^a^	12.63 ± 1.78^a^	3.99 ± 0.63^a^

Values were expressed as means ± SD (*n* = 39). Data within the same column of each group bearing different superscript letters were significantly different (*p* < 0.05).

Elevated ROS generation highly affect liver (hepatocytes) cell as they utilize more ATP via electron transport chain (ETC) cycle, as most of the ROS are generated during ETC cycle, hence hepatocytes are highly prone to free-radical-induced damage. Increased ROS trigger lipid peroxidation in hepatocytes and leads to decreased hepatocytes content as well as altered metabolism (lowering detoxifying enzymes and endogenous antioxidant enzymes) and thereby hasten the oxidative damage by increasing oxidative stress. GOT (AST) and GPT (ALT) were the most reliable hepatic markers for assessing the levels of hepatic damage (Wu et al. 2016). The hepatic marker enzymes in plasma of placebo and GL-treated healthy subjects are epitomized in Table 4. The substantial decrement in the activities of GOT and GPT was observed in GL consumed subjects in the comparison between baseline and the end of the intervention, thus inferred that GL probably lowered oxidative stress and thereby lowering hepatic damage and hence improved hepatic function by lowering marker enzymes. Shi et al. (2008) proved that GL could act as a hepatoprotective agent against d-galactosamine-induced liver injury in a mouse model. Recently, Liu et al. (2014) showed that some triterpenoids of *Ganoderma* species exhibited hepatoprotective activities by successfully scavenging free radicals. No significant modification was observed in the placebo-treated group from baseline to sixth month, in any of the biochemical parameters.

**Table 4.** The hepatic marker enzymes in plasma of GL and placebo-treated healthy subjects.

	Group	GPT (U/L)	GOT (U/L)
Baseline	GL	22.58 ± 5.13^a^	20.65 ± 4.37^a^
	Placebo	20.05 ± 4.93^a^	19.65 ± 5.46^a^
Third month	GL	16.85 ± 3.46 ^b^	19.85 ± 4.63^a^
	Placebo	20.25 ± 3.94^a^	20.45 ± 4.74^a^
Sixth month	GL	13.08 ± 2.79^c^	15.50 ± 3.17^b^
	Placebo	21.33 ± 5.11^a^	19.90 ± 4.83^a^

Values were expressed as means ± SD (*n* = 39). Data within the same column of each group bearing different superscript letters were significantly different (*p* < 0.05).

**Figure 2.**
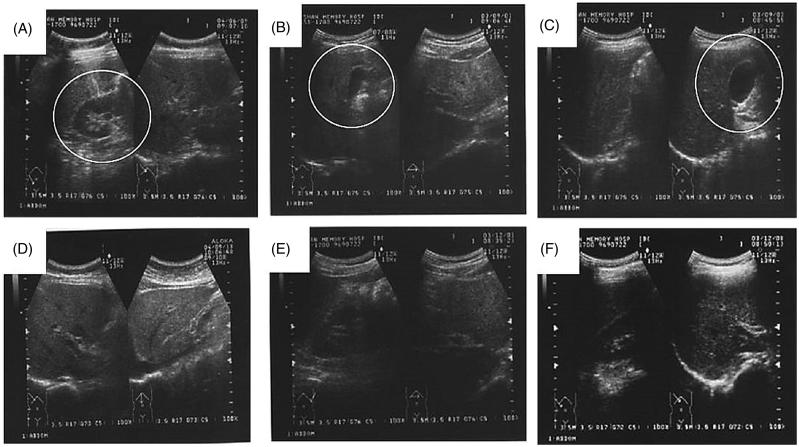
The abdominal ultrasonic image of GL-treated healthy subjects. Image A, B and C represents subject no. 10, 19 at baseline with mild fatty liver and subject no. 36 at baseline with gall bladder polyp (indicated with a circle). Image D, E and F represents subject no. 10, 19 and 36 after 6 months of GL treatment display normal hepatic structure without any signs of fatty liver or gallbladder polyp.

The abdominal ultrasonic examination of GL-treated subjects is represented in Figure 2, which revealed the conditions of mild fatty liver (Figure 2(A,B)) and gall bladder polyp (Figure 2(C)) in the subject no.10, 19 and 36, respectively, at baseline. Administration of GL for 6 months on those subjects became normal with no signs of fatty liver (Figure 2(D,E)) and gall bladder polyp (Figure 2(F)). Moreover, Chu et al. (2012) in a randomized clinical trial inferred that owing to the presence of triterpenoids, which are similar to lanosterol in GL might contribute to lipid-lowering property. Thus, lipid accumulation in the form of fatty liver can be regressed to normal by its lipid-lowering activity. The results of the hepatic markers and abdominal ultrasound examination indicated that GL could improve hepatic function (hepatoprotective) by reducing the oxidative stress (ROS), lipid deposition and thereby lowers the hepatic damage as well as normalize various abnormal conditions such as fatty liver and gallbladder polyp.

Limitation of the present study is the involvement of a small number of subjects, and the inclusion of only mild liver dysfunctional subjects thus made this trial inconclusive. In future, a significant number of moderate or severe liver dysfunctional subjects might be used to justify our results. To date, no universally accepted aging biomarker have been identified and hence, we evaluated some commonly accepted ubiquitous oxidative stress biomarkers such as TBARS and 8-OH-dG as well as various anti-oxidant markers such as TEAC, thiols, SOD, CAT, Gpx, GSH and GR. Furthermore, determining the anti-aging process in a human system is a tedious process, since several factors might have an indirect impact on human health and the aging process (Pandey & Rizvi 2010).

## Conclusions

The results of the present study in GL-treated subjects revealed a substantial reduction in the levels of various oxidative stress markers as well as a concomitant escalation in the levels of various anti-oxidative status from the initial phase (baseline) to the final phase (6 months). Moreover, the hepatic damage and morphology were normalized with the treatment with GL. Thus, we concluded that GL enriched with triterpenoids and polysaccharide (synergic effect) might influence antioxidative, anti-aging and hepatoprotective efficacy via attenuating overproduction of free radicals and thereby protecting the cell from damage. In future, the active components of GL (triterpenoids and polysaccharide) are used to examine the exact molecular mechanism for antioxidation, anti-aging and hepatoprotection with many moderate or severe liver dysfunctional subjects.
